# Complex endocarditis in a young patient

**DOI:** 10.1093/ehjimp/qyae132

**Published:** 2024-12-19

**Authors:** Marie-Luise Dikou, Abigail Gowland, Sara Volpi, Julia Grapsa, Gianluca Lucchese

**Affiliations:** Cardiology Department, Guy’s and St Thomas’ NHS Foundation Trust, Westminster Bridge, SE1 7EH London, United Kingdom; Cardiology Department, Guy’s and St Thomas’ NHS Foundation Trust, Westminster Bridge, SE1 7EH London, United Kingdom; Department of Cardiothoracic Surgery, Guy’s and St Thomas’ NHS Foundation Trust, London, United Kingdom; Cardiology Department, Guy’s and St Thomas’ NHS Foundation Trust, Westminster Bridge, SE1 7EH London, United Kingdom; Department of Cardiothoracic Surgery, Guy’s and St Thomas’ NHS Foundation Trust, London, United Kingdom

**Keywords:** infective endocarditis, valvular heart disease, surgery, echocardiography

A 36-year-old female patient presented to her local district general hospital feeling breathless, generally unwell, and lethargic on a background of a complex past medical history including an episode of Libman–Sacks endocarditis a year previously. She also had systemic lupus erythematosus with lupus nephritis and end-stage renal disease on haemodialysis, antiphospholipid syndrome, thrombocytopaenia, and recurrent pulmonary haemorrhages. She had previously undergone plasma exchange and rituximab treatment and was now on cyclophosphamide.

During her previous endocarditis episode and following a multidisciplinary meeting, with the main concern the previous alveolar haemorrhages, she was judged high risk for open-heart surgery and underwent a transcatheter aortic valve replacement (26 mm S3) locally.

The patient was transferred to our tertiary centre on this presentation with signs of heart failure, multiple positive blood cultures for *Staphylococcus epidermidis*, septic pulmonary emboli, and suspicion of aortic root abscess on transthoracic echocardiogram (*Videos 1* and *2*).

On presentation, she was clinically septic, had an infected indwelling permanent line, and grew *S. epidermidis* on repeat blood cultures, which was discussed with microbiology and treated with a course of vancomycin and rifampicin.

She was investigated with multiple imaging modalities including transthoracic and transoesophageal echocardiogram. She was discovered to have a large aortic root abscess (*Figure 1*, red arrow) with associated vegetation and fistulation into the right atrium (green arrow), as well as posterior mitral annular abscess (yellow arrow).

**Figure 1:**
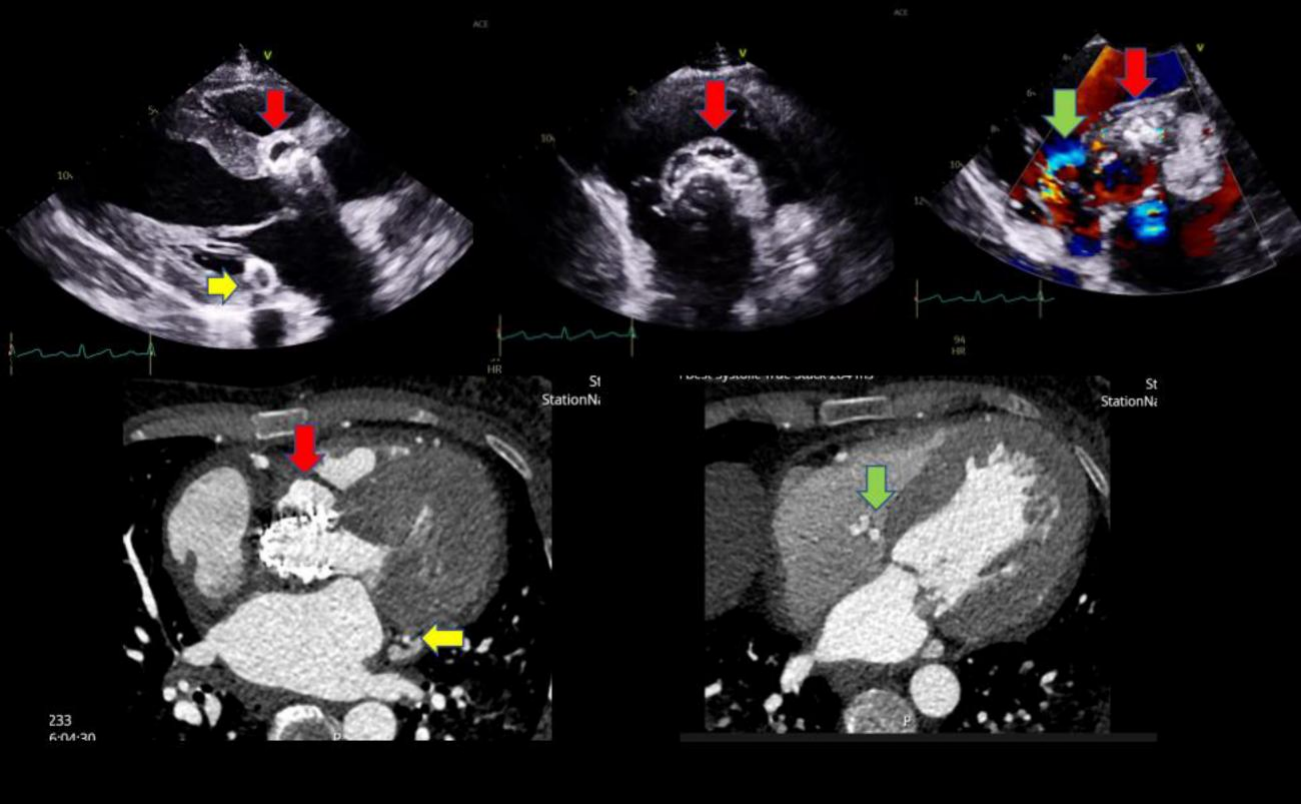
Multipanel figure demonstrating three different pathologies identified on the patient—upper panel is the transthoracic echocardiogram and lower panel the ECG-gated computed tomography scan. Red arrow demonstrates the aortic root abscess, yellow arrow the posterior mitral annulus abscess, and green arrow the fistula from the left ventricular outflow tract to the right atrium.

Despite the high surgical risk and after extensive discussion with the heart multidisciplinary care team, the patient underwent open-heart surgery without the need of extracorporeal support, with a good recovery.

## Notes

**Funding:** None declared.

**Consent:** We have obtained written consent of the patient.

**Data availability:** No data for statistical review.

## Lead author biography

Dr Marie-Luise Dikou is an internal medicine trainee in South London. She has previously completed junior clinical fellowships in cardiology and emergency medicine at St Thomas’ hospital and wishes to pursue cardiology specialty training in the future. She is currently a successful CLMIB grant recipient for the prevention programme and a junior reviewer for the *European Heart Journal - Case Reports*.

